# Trigger videos: a novel application of a tool for surgical faculty development

**DOI:** 10.1186/s12893-021-01415-9

**Published:** 2021-12-17

**Authors:** Anuj Arora, Jen Hoogenes, Deepak Dath

**Affiliations:** 1 Division of General Surgery, Department of Surgery, University of Toronto, Toronto, ON Canada; 2 Department of Surgery, McMaster University, Hamilton, ON Canada; 3 Division of General Surgery, Department of Surgery, McMaster University, Hamilton, ON Canada; 4 Division of General Surgery, Department of Surgery, Juravinski Hospital and Cancer Centre, 711 Concession Street, Hamilton, ON L8V 1C3 Canada

**Keywords:** Trigger videos, Faculty development, Intraoperative teaching, Learning tool

## Abstract

**Background:**

Trigger videos have occasionally been used in medical education; however, their application to surgical faculty development is novel. We assessed participants’ attitudes towards workshops on intraoperative teaching (IOT) that were anchored by trigger videos, and studied whether they could generate discussion-for-learning among surgeons in this workshop setting.

**Methods:**

Surgeons from multiple specialties attended one of six faculty development workshops where IOT trigger videos were shown and discussed during break-out sessions. Participants completed questionnaires to (1) evaluate videos via survey and feedback, and (2) identify adoptable and discardable IOT techniques. Teaching techniques were collated to identify planned IOT changes and survey data and feedback were analyzed.

**Results:**

A total of 135 surgeons identified 292 adoptable and 202 discardable IOT techniques based on trigger videos and discussions, and 94% of participants reported that the trigger videos were useful and encouraged them to discuss and consider new IOT techniques in their own practice.

**Conclusions:**

Participants reported that the trigger videos were useful and motivating. Surgeons critically reflected on IOT during the sessions, identifying numerous adoptable and discardable techniques relevant to their own teaching styles. Trigger videos can be a valuable tool for surgical faculty development and can be tailored to other medical specialties.

**Supplementary Information:**

The online version contains supplementary material available at 10.1186/s12893-021-01415-9.

## Background

Academic surgeons must actively teach, adapt teaching techniques while considering trainees’ learning needs, entrust residents with increasing intraoperative autonomy, and provide residents with meaningful feedback [1]. Yet, surgeons teach with little to no formal training in adult education and teaching [2, 3]. Intraoperative teaching (IOT) can be especially challenging, as it occurs in the complex environment of the operating room (OR) where the surgeon is also responsible for the patient’s outcome and where resident education is secondary to patient safety [2]. Surgical training programs are shifting towards competency-based medical education (CBME) frameworks which raises new challenges for educators. In this new CBME era, frequent assessment, feedback, and documentation means that surgeons need to be constantly mindful of how they are teaching in the OR [1].

IOT requires surgeons to develop a complicated skillset; but it is uncommon for them to have time to reflect on or review their teaching skills with colleagues to know how to improve [4]. In 2018, Deal and colleagues identified several key priorities for faculty development in general surgery, which included feedback and assessment of residents and the improvement of IOT skills [5]. Holding regular faculty development sessions that focus on teaching and assessment can provide surgeons with the skills and training required to teach effectively.

Faculty development in the setting of an OR would be ideal, but this is not possible in surgery given the scale on which faculty development must be delivered. Current techniques include small-group discussions, interactive exercises, structured opportunities for reflection, didactic lectures, role-play and simulation, films, and videotaped reviews of performance [6]. Immersive techniques and sessions that are closer to real-life experiences often result in the most effective faculty development outcomes [6]. For general surgery, Deal et al. found that the most beneficial learning modalities are interactive small group sessions and video-based education, noting that fundamental barriers include time limitations, faculty disinterest, and limited financial support for new faculty development initiatives [5]. With these considerations, we delivered faculty development sessions that capitalized on video-based education to provide a realistic and immersive experience.

Our team designed IOT faculty development workshops around a less commonly used tool known as a trigger video. Trigger videos are short, realistic, challenging, or routine scenarios that are meant to stimulate meaningful discussion and reflection among faculty [7, 8]. Trigger videos are not “how-to” or “show how” teaching material for learning how to do a particular operation; rather, workshop participants are meant to become immersed in the teaching scenarios that depict operations with trainees. The videos therefore provide a common experience from which the participants in small groups can generate discussions about how to teach, regardless of the specialty of the participants. The scenarios also encourage reflection with the goal of yielding improved results over passive learning techniques [9].

The available literature supporting trigger videos has outlined advantages for medical students, nurses and medical educators. In problem-based learning sessions, trigger videos have been highly rated by students as being engaging and motivating [9]. In nurse education programs, trigger videos have been shown to be excellent stimuli for discussion and analysis of complex issues [10]. Additionally, nurse anesthesia instructors have noted that trigger videos help to hone skills necessary to teach in high stress environments [11]. For medical educators, a pilot of a video-based faculty development curriculum showed promise to affect change in teaching practices [12]. We employed trigger videos in our workshops to exploit these advantages for faculty development in surgery.

We hypothesized that workshops anchored by trigger videos would provide surgical faculty with an immersive experience with their peers that would facilitate learning about how to improve their IOT. To study this, we evaluated the attitudes towards workshops designed with trigger videos and whether they were able to generate discussion about how participants could make changes to improve their IOT techniques. Here, we describe the development of our trigger videos, the design of our workshops, the analysis of the participants’ evaluations, and the potential applications for future faculty development initiatives.

## Methods

### Ethics approval

This research project was exempt from research ethics board review by the Hamilton Integrated Research Ethics Board [HIREB] given that this work was considered quality improvement and program evaluation. Informed consent was obtained by participants attending the workshop and filling the feedback forms. All methods were carried out in accordance with relevant guidelines and regulations.

### Study design and setting

In a previous faculty development study, 44 experienced staff surgeons from different specialties participated in focus group sessions designed to initiate discourse with respect to challenges faced during IOT [13]. These sessions identified multiple IOT topics which were used to lay the foundation for the trigger videos [13]. Surgical educators were invited to develop scenarios and scripts for highlighting the different IOT topics to create the videos. The videos were produced using volunteer medical students, residents, faculty, nurses, and other hospital personnel in realistic, simulated settings in the OR. Table 1 lists the five trigger videos that were designed to highlight different IOT challenges that could be faced in a variety of surgical specialties.

**Table 1:** Trigger videos used in the faculty development sessions

Trigger video	Description	Teaching challenges/behaviors depicted*
Open gastrectomy	Trigger video depicting teaching challenges faced by a staff surgeon during a complex case (OR running late risking cancellation of last case, intraoperative bleeding, surgeon worried about her car)	Managing time pressures, personal, and OR-related distractions
Retroperitoneal node dissection	Trigger video depicting negative and positive teaching strategies to cope with significant intraoperative bleeding	Losing composure during a case, failing to lead, blaming resident, unclear communication with OR team, assisting resident and role-modeling in a difficult situation
Right hemicolectomy	Trigger video depicting an intern’s first day on a service where the staff and senior resident are not interested in teaching and the intern is “getting in the way”	Teaching to only one level of learner, poor role modeling, inappropriate behavior in OR, negative teaching environment
Laparoscopic cholecystectomy	Trigger video depicting two different strategies to coach a resident on a type of case the resident had previously struggled with, while also teaching the medical student	Teaching multiple levels of learners, providing feedback and instruction when resident struggles, debriefing after a case
Ankle open reduction and internal fixation (ORIF)	Trigger video depicting a resident struggling through a case without getting instruction or feedback from the staff surgeon	Inability to adapt to level of learner, poor role modeling, failure to brief before case, being distracted as a teacher

*Some teaching challenges built into the videos are listed above, but many more nuanced issues were identified by participants as shown in Table 2

The trigger videos anchored three types of workshops for a total of six events. The first type was at our university where all surgeons in the department were invited (three events). The second type was held by invitation at another university where surgeons from a specific division or hospital site were participants (two events), while the third type was conference-based and was attended by surgeons from a variety of disciplines (one event). Workshops were one to three hours long in duration depending on venue and allotted time.

Workshop participants were assigned to small groups by tables and all participants were shown the trigger video as a large group. During small group break-out sessions, participants at their tables were instructed to discuss the IOT techniques and concepts they identified in the video. Each small group then presented a summary of what they learned when the workshop reconvened in a large group discussion format. Workshops ended with a facilitated discussion by a workshop leader who linked the content the participants discussed with evidence from the literature. One or two trigger videos were used during the sessions, depending on the duration of the workshop. At the end of the workshop, participants filled the evaluation and feedback forms.

### Outcomes

**Fig. 1:**
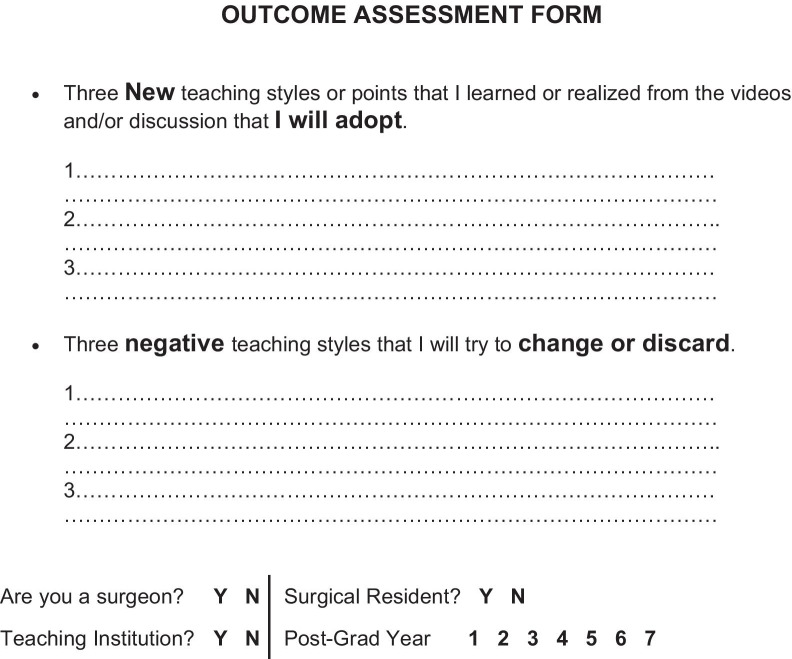
Positive and negative teaching styles questionnaire

**Fig. 2:**
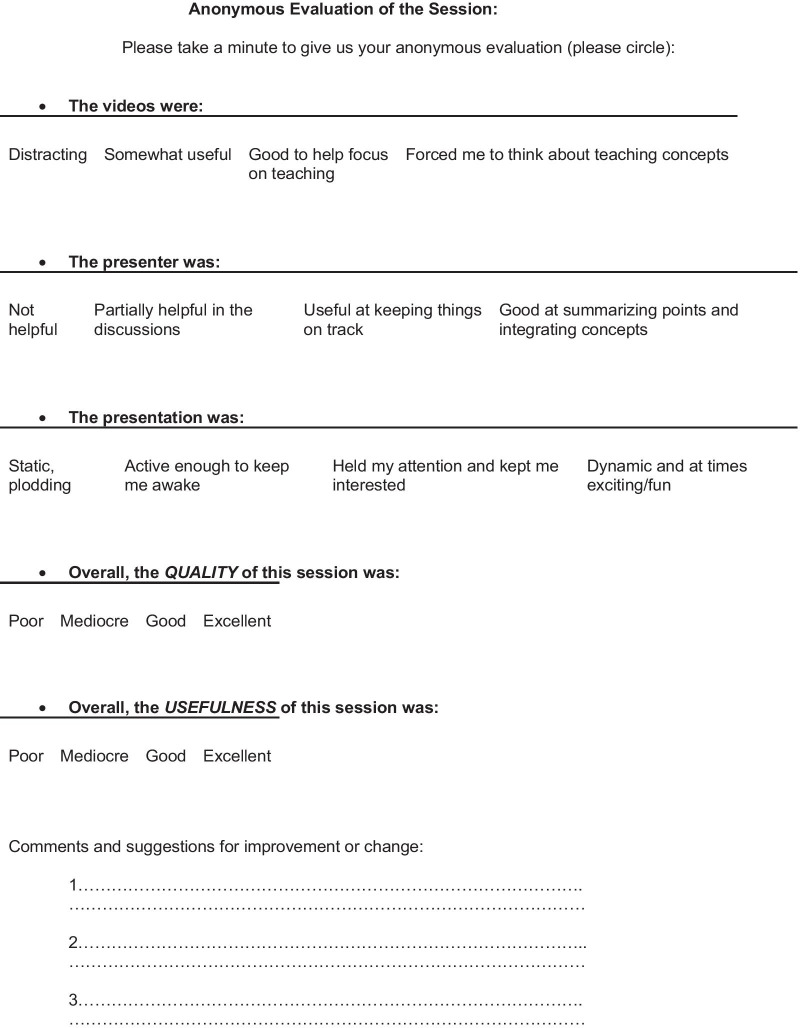
Trigger video evaluation questionnaire

The outcomes for this study included evaluating whether the workshop engaged participants and whether trigger videos encouraged participants to identify techniques to improve their own IOT. For evaluation of the workshops, participants completed a Likert-style survey that elicited feedback on the presentation, the quality and utility of the session, and their opinion of the trigger videos. Each question had four different response options reflecting two positive and two negative attitudes. An open-ended question was included for comments and suggested improvements for future application. To evaluate whether the participants planned to change their IOT techniques, we asked them to report three positive techniques they would adopt and three negative techniques they would discard based on their workshop discussions. The evaluation forms are shown in Figs. 1 and 2.

### Analysis

To evaluate the effectiveness of engagement of the trigger videos, the questionnaire data were dichotomized into positive and negative responses and reported in a dichotomized fashion. The quotes from the comments section were analyzed using a standard qualitative content analysis approach by all three authors [14]. Key descriptive content was extracted and then responses were compiled into three categories: positive comments, suggestions for innovation, and critical comments. To assess whether the participants planned to adopt or discard IOT techniques, the positive and negative techniques from the questionnaires were collated.

## Results

The workshops were attended by surgeons from multiple specialities (general surgery, plastic surgery, orthopedic surgery, urology, gynecology, cardiac surgery, pediatric surgery, neurosurgery, vascular surgery, thoracic surgery, head and neck surgery, ophthalmology). A total of 119 participants completed the Likert-type questionnaire evaluating the trigger videos, with 94% indicating that the videos were “good” or “forced me to think about teaching concepts” while 6% thought the videos were “distracting” or “somewhat useful”. Almost all (95%) respondents thought the presentation was “dynamic and at times exciting and fun” or “held my attention and kept me interested”, compared to 5% of respondents who thought it was “static, plodding” or “[just] active enough to keep me awake”. Regarding usefulness of the workshop, 97% of thought the session was “good” or “excellent”, while 3% felt it was “poor” or “mediocre”. A total of 98% rated the workshop’s quality as “good” or “excellent”, with the remainder responding that it was “poor” or “mediocre”. Full questionnaire data is highlighted in Additional file 2: Appendix 2.

There were a total of 58 comments, 20 were positive, mostly complementing on the delivery of the workshop, and included quotes like “[the] session will actually affect my behavior” and “great forum for all faculty to open up about teaching issues”. Surgeons suggested 21 innovations for practice, including “emphasizing [the] CanMEDS framework” and “including nurses, anesthesiologists, and residents [in their IOT]”. Critical feedback was noted in 17 comments that indicated the workshops were “too long”.

The teaching techniques questionnaire was completed by 135 surgeons, where 298 positive “adoptable” and 202 negative “discardable” teaching styles were identified. Example quotes are shown in Table 2.

**Table 2:** Quoted examples generated during discussion after viewing trigger videos of positive IOT styles surgeons wanted to adopt and negative styles they wanted to discard

Positive “adoptable” teaching styles	Negative “discardable” teaching styles
∙ Make sure to remain interactive with the junior trainee during a case ∙ Continuous questioning to all learners in O.R. ∙ Empower resident to control and participate in the environment ∙ Avoid outside stresses ∙ Being better assistant (not getting distracted) ∙ Anticipate potential problems and situations ∙ Label my behaviour to resident (i.e., CanMEDS) ∙ Emphasize the professional role with regards to setting the tone in the OR ∙ Breakdown common cases into teachable components ∙ Quick chat to plan the steps of the surgery with the resident ∙ Explain decisions in OR ∙ Try to talk to residents more through difficult parts rather than take over ∙ Identify verbally, i.e., voice ‘learning moment’ ∙ Outline expectations for different levels of learners ∙ Delegate different roles to different levels of training ∙ Let resident choose what to do if there is time constraint ∙ Reminder to time and book OR cases when working with trainees ∙ Asking resident to provide feedback to you as a teacher ∙ Better use of feedback/debriefing after case ∙ Invite feedback from trainees ∙ Pre- and post-case discussion with residents ∙ Understanding learner needs/expectations ∙ Be more explicit about key learning objectives for case ∙ Debrief about case post-op ∙ Go over teaching points	∙ Not engaging in the training or teaching ∙ Not promoting resident self-confidence ∙ Non-case-based discussion that may distract ∙ Being distracted by personal life issues ∙ Allowing frustration with sub-optimal instruments to affect mood/tone in OR ∙ Not speaking up for others ∙ Not advocating for trainees ∙ Not being polite to nursing staff ∙ No teaching plan for OR ∙ Allowing unprepared residents to proceed to OR ∙ Unprepared (to teach) ∙ Assuming residents know what I know/next steps ∙ Failure to communicate the thought process ∙ Poor communication with other members of the OR team ∙ Taking over with no explanation ∙ Ignoring medical students while teaching residents ∙ Minimizing role of junior learners/medical students ∙ Hierarchical downplay ∙ Projecting feelings of being rushed ∙ Thinking too much about time pressures ∙ More patience before taking over ∙ Silence—not giving feedback ∙ Eliminate negative banter, teasing or ridicule ∙ Criticism in OR that may embarrass resident ∙ Not making more time for feedback ∙ Blaming the learner ∙ Not debriefing at the end of case ∙ Not talking more pre/post and during case

## Discussion

In this prospective cross-sectional study, we designed, delivered, and evaluated faculty development workshops for surgeons that used trigger videos as a tool to improve IOT. The participants were engaged and stimulated during their discussions amongst their peers in both the large and small group formats. Feedback from participants was overwhelmingly positive with respect to the use of trigger videos as a cornerstone of these sessions. A total of 500 unique IOT points were generated by participants who completed the post-workshop questionnaires, indicating that they were able to relate the workshop content to their own teaching experiences.

A barrier to faculty development in surgery is interest [5]. The use of videos in medical education has been used to overcome this barrier, as they can encourage interactivity to improve learning [15]. For simple content, "how-to" instructional videos are excellent at showing or explaining concepts. For complex content where participants need to remain engaged and interactive, trigger videos are indispensable for generating discussion among peers. Our results show that the trigger videos used in our workshops are capable of inciting dialogue while keeping participants engaged. Positive feedback on the evaluation questionnaires showed that the participants enjoyed using the videos and their open-ended responses demonstrated that the videos generated reflection, discussion, and plans for behaviour change.

Our finding that trigger videos can be an excellent stimulus for discussion has been replicated in other studies. Ber and Alroy used trigger videos to teach aspects of professionalism to medical students, finding that participants identified a multitude of issues relative to the topic [16]. In our study, participants were able to identify unique issues and challenges they faced in their own daily teaching practice. Despite viewing the same videos, each group highlighted a variety of discussion points related to IOT. This allowed for rich discussion in the small groups, which set the foundation for broader large group discourse. This is similar to Clement’s study where general practice medical educators were able to use videos of their teaching practices as stimulus for discussion for improving teaching [12]. Ber and Alroy also found that medical students’ perspectives on professionalism differed when viewing trigger videos prior to clerkship compared to viewing the videos after initiating clinical experience, indicating that the same trigger video format can be used at different stages of training [16]. Participants in our workshops had varying levels of teaching experience and were able to learn from the trigger videos and from their peers. In a study by Nichols, when used in nursing education, trigger videos were noted to be excellent discussion stimulators and were rated as highly enjoyable among the students [10]. These findings parallel the results of our study and also suggest that trigger videos can be successful in multiple educational settings.

This paper describes a novel application of trigger videos in the setting of faculty development workshops where surgical faculty learn about IOT. Coaching, small group sessions, and video-based education have been highlighted as the top three learning modalities for faculty development [5]. Our video-based workshops with small group discussion capitalized on two of these techniques. Using the trigger videos as an anchor, participants were able to identify and discuss the IOT challenges they face in their own daily practice and strategies to tackle them. Participants were also able to learn from their peers, as each small group focused on specific points introduced in the trigger videos. They shared by reporting the elements of their small group discussions to the larger group, benefiting the entire workshop. The facilitators presented literature that supported the IOT techniques to further solidify what the surgeons had learned during these interactive workshops.

The trigger video is a successful tool because it is immersive. It uses active learning techniques to improve retention of material, encourage motivation for further study, and develop new thinking skills [17]. We believe that the trigger video works by forcing participants to debrief their immersive experiences, allowing for deep reflection and critical thinking among peers in a non-judgemental environment. Debriefing is cited as one of the most important aspects of learning because it translates an experience into an analyzed and interpreted event [18]. In our faculty development workshops for surgeons, deep reflection and critical thinking during discussion with peers caused participants to evaluate their own IOT styles and behaviours and how they intend to change the way they teach. Based on participants’ feedback and identification of 500 unique IOT concepts, we believe trigger videos were a highly valuable component of our faculty development workshops.

Barriers to faculty development include financial support for implementing workshops [5]. A trigger video is an inexpensive tool that can be used multiple times. Videos are ubiquitous, can be produced easily (sophisticated smartphone cameras, inexpensive microphones, and free editing software are readily available) or found online (including those we developed for our workshops, available in Additional file 1: Appendix 1). Additionally, video can easily be shown online and on virtual platforms, making trigger videos an attractive solution for engaging participants during faculty development initiatives on virtual platforms which is important as we emerge from COVID-19 [19]. For example, a trigger video can be shown to a large online group, followed by virtual breakout “rooms” for discussion, and then reconvening in a large group format virtually.

Our study’s strengths include the vast acceptance of the trigger videos by the participants in the workshops, the demonstration that trigger videos are an engaging tool that can generate discussion about complex concepts such as IOT, and the potential for generalizability from local to multi-institutional delivery of the workshops with a variety of surgeons. The study is limited in that our data may be skewed by participation bias, as participants who chose to attend these faculty development sessions may already have an interest in teaching. We also had no control group for comparison. Furthermore, scheduling of faculty development workshops is a barrier for some, as is the case with many scheduled teaching initiatives. However, with the potential for the use of workshops with trigger videos on a virtual platform, this could alleviate scheduling conflicts for those who wish to participate.

Surgical faculty development initiatives should focus on training participants for their new teaching and assessment roles in the CBME era [5]. To ensure their residents and fellows attain and demonstrate competency, surgeons need to focus on their own IOT skills. In our study, small-group sessions using trigger videos were effective at encouraging participants to focus on IOT skills. Our trigger videos are available free online (Additional file 1: Appendix 1); but trigger videos can be created using ideas from existing videos, filmed to fit the needs of a workshop or other teaching initiative, and can be used on virtual platforms. Given our success with implementing trigger videos, we believe that strong consideration should be given to using this tool to anchor faculty development sessions focused on IOT. Furthermore, given that trigger videos can be tailored to multiple scenarios, they may be a useful component for other types of teaching modalities and transferable to other medical specialties.

## Conclusions

This paper describes a novel application of trigger videos in the setting of surgical faculty development workshops designed for educating and improving IOT techniques. Incorporating trigger videos in our six workshops allowed for rich discussion and demonstrated that trigger videos are engaging tools that can help to facilitate small and large group dialogue on complex concepts such as IOT. Participants’ positive feedback and their stated intentions to improve their IOT techniques indicates the value of using trigger videos in a faculty development setting. The use of trigger videos for IOT may be adapted from local to multi-institutional delivery of faculty development workshops and may be a feasible option for use via online platforms and with other medical specialties.

## Supplementary Information

**Additional file 1: Appendix 1.** Trigger video YouTube® links.

**Additional file 2: Appendix 2.** Likert-style questionnaire data for evaluation of; the trigger videos (**A**), presentation (**B**), quality of the session (**C**), and usefulness of the session (**D**).

## Data Availability

The datasets used and/or analysed during the current study are available from the corresponding author on reasonable request.

## Abbreviations

IOT: Intraoperative teaching
CBME: Competency-based medical education

## References

[1] Sonnadara RR, Mui C, McQueen S, Mironova P, Nousiainen M, Safir O, Kraemer W, Ferguson P, Alman B, Reznick R (2014). Reflections on competency-based education and training for surgical residents. J Surg Educ.

[2] Iwaszkiewicz M, DaRosa DA, Risucci DA (2008). Efforts to enhance operating room teaching. J Surg Educ.

[3] Jeffree RL, Ten CRM (2010). tips for teaching in the theatre tearoom: shifting the focus from teaching to learning. World J Surg.

[4] Khan N, Khan MS, Dasgupta P, Ahmed K (2013). The surgeon as educator: fundamentals of faculty training in surgical specialties. BJU Int.

[5] Deal SB, Alseidi AA, Chipman JG, Gauvin J, Meara M, Sidwell R, Stefanidis D, Schenarts PJ (2018). Identifying priorities for faculty development in general surgery using the Delphi consensus method. J Surg Educ.

[6] Steinert Y, Mann K, Anderson B, Barnett BM, Centeno A, Naismith L, Prideaux D, Spencer J, Tullo E, Viggiano T, Ward H, Dolmans D (2016). A systematic review of faculty development initiatives designed to enhance teaching effectiveness: a 10-year update: BEME guide No. 40. Med Teach.

[7] Fisch AL (1972). The trigger film technique. Improv Coll Univ Teach.

[8] Ber R, Alroy G (2001). Twenty years of experience using trigger films as a teaching tool. Acad Med.

[9] Chan LK, Patil NG, Chen JY, Lam JCM, Lau CS, Ip MSM (2010). Advantages of video trigger in problem-based learning. Med Teach.

[10] Nichols J (1994). The trigger film in nurse education. Nurse Educ Today.

[11] Hartland W, Biddle C, Fallacaro M (2003). Accessing the living laboratory: trigger films as an aid to developing, enabling, and assessing anesthesia clinical instructors. Am Assoc Nurse Anesth.

[12] Clement T, Howard D, Lyon E, Silverman J, Molloy E (2020). Video-triggered professional learning for general practice trainers: using the “cauldron of practice” to explore teaching and learning. Educ Prim Care.

[13] Dath D, Hoogenes J, Matsumoto ED, Szalay DA (2013). Exploring how surgeon teachers motivate residents in the operating room. Am J Surg.

[14] Qualitative Research & Evaluation Methods | SAGE Publications Inc. https://us.sagepub.com/en-us/nam/qualitative-research-evaluation-methods/book232962. Accessed 30 Oct 2020.

[15] Dong C, Goh PS (2015). Twelve tips for the effective use of videos in medical education. Med Teach.

[16] Ber R, Alroy G (2002). Teaching professionalism with the aid of trigger films. Med Teach.

[17] Charles C, James A, Bonwell CC, Eison JA. Active learning: creating excitement in the classroom. ASH#-ERIC Higher Education Report No. 1. Washington, D.C.: The George Washington University, School of Education and Human Development; 1991.

[18] Fanning RM, Gaba DM (2007). The role of debriefing in simulation-based learning. Simul Healthc.

[19] Doulias T, Gallo G, Rubio-Perez I, Breukink SO, Hahnloser D (2020). Doing more with less: surgical training in the COVID-19 era. J Invest Surg.

