# Supplementary material for: Trigger videos: a novel application of a tool for surgical faculty development
Source: BMC Surg. 2021 Dec 17;21:424. doi: 10.1186/s12893-021-01415-9 (PMC8680058; doi:10.1186/s12893-021-01415-9)
Supplement: Supplementary file 1 — Additional file 1: Appendix 1. Trigger video YouTube® links. [file 12893_2021_1415_MOESM1_ESM.docx]

**Appendix 1.** Trigger video YouTube® links

| **Video name** | **Link** |
| --- | --- |
| Open gastrectomy | https://www.youtube.com/watch?v=825ldhM7fUk&t=11s |
| Retroperitoneal node dissection | https://www.youtube.com/watch?v=Lc5PD_5if_s&t=25s |
| Right hemicolectomy | https://www.youtube.com/watch?v=6kytI3ETBHs&t=1s |
| Laparoscopic cholecystectomy | https://www.youtube.com/watch?v=bGv-Q-XWTIw&t=7s |
| Ankle ORIF | https://www.youtube.com/watch?v=NLzAGjrjK1o&t=8s |
