# Supplementary material for: Trigger videos: a novel application of a tool for surgical faculty development
Source: BMC Surg. 2021 Dec 17;21:424. doi: 10.1186/s12893-021-01415-9 (PMC8680058; doi:10.1186/s12893-021-01415-9)
Supplement: Supplementary file 2 — Additional file 2: Appendix 2. Likert-style questionnaire data for evaluation of; the trigger videos (A), presentation (B), quality of the session (C), and usefulness of the session (D). [file 12893_2021_1415_MOESM2_ESM.docx]

C

D

B

A

**Appendix 2:** Likert-style questionnaire data for evaluation of; the trigger videos (A), presentation (B), quality of the session (C), and usefulness of the session (D).
